# Probiotics and Synbiotics Addition to Bama Mini-Pigs' Diet Improve Carcass Traits and Meat Quality by Altering Plasma Metabolites and Related Gene Expression of Offspring

**DOI:** 10.3389/fvets.2022.779745

**Published:** 2022-07-05

**Authors:** Qian Zhu, Mingtong Song, Md. Abul Kalam Azad, Cui Ma, Yulong Yin, Xiangfeng Kong

**Affiliations:** ^1^Hunan Provincial Key Laboratory of Animal Nutritional Physiology and Metabolic Process, Institute of Subtropical Agriculture, Chinese Academy of Sciences, Changsha, China; ^2^University of Chinese Academy of Sciences, Beijing, China; ^3^Research Center of Mini-Pig, Huanjiang Observation and Research Station for Karst Ecosystems, Chinese Academy of Sciences, Huanjiang, China

**Keywords:** Bama mini-pigs, carcass traits, growth performance, meat quality, plasma metabolites, probiotics, synbiotics

## Abstract

This study evaluated the effects of maternal probiotics and synbiotics addition on several traits and parameters in offspring. A total of 64 Bama mini pigs were randomly allocated into the control (basal diet), antibiotic (50 g/t virginiamycin), probiotics (200 mL/day probiotics), or synbiotics (500 g/t xylo-oligosaccharides and 200 mL/day probiotics) group and fed with experimental diets during pregnancy and lactation. After weaning, two piglets per litter and eight piglets per group were selected and fed with a basal diet. Eight pigs per group were selected for analysis at 65, 95, and 125 days of age. The results showed that the addition of probiotics increased the average daily feed intake of the pigs during the 66- to 95-day-old periods and backfat thickness at 65 and 125 days of age, and that the addition of synbiotics increased backfat thickness and decreased muscle percentage and loin-eye area at 125 days of age. The addition of maternal probiotics increased the cooking yield and pH_45min_ value at 65 and 95 days of age, respectively, the addition of synbiotics increased the meat color at 95 days of age, and the addition of probiotics and synbiotics decreased drip loss and shear force in 65- and 125-day-old pigs, respectively. However, maternal antibiotic addition increased shear force in 125-day-old pigs. Dietary probiotics and synbiotics addition in sows' diets increased several amino acids (AAs), including total AAs, histidine, methionine, asparagine, arginine, and leucine, and decreased glycine, proline, isoleucine, α-aminoadipic acid, α-amino-n-butyric acid, β-alanine, and γ-amino-n-butyric acid in the plasma and *longissimus thoracis* (LT) muscle of offspring at different stages. In the LT muscle fatty acid (FA) analysis, saturated FA (including C16:0, C17:0, and C20:0) and C18:1n9t contents were lower, and C18:2n6c, C16:1, C20:1, and unsaturated FA contents were higher in the probiotics group. C10:0, C12:0, and C14:0 contents were higher in 65-day-old pigs, and C20:1 and C18:1n9t contents were lower in the synbiotics group in 95- and 125-day-old pigs, respectively. The plasma biochemical analysis revealed that the addition of maternal probiotics and synbiotics decreased plasma cholinesterase, urea nitrogen, and glucose levels in 95-day-old pigs, and that the addition of synbiotics increased plasma high-density lipoprotein cholesterol, low-density lipoprotein cholesterol, and total cholesterol concentrations in 65-day-old pigs and triglyceride concentration in 125-day-old pigs. The addition of maternal probiotics and synbiotics regulated muscle fiber type, myogenic regulation, and lipid metabolism-related gene expression of LT muscle in offspring. In conclusion, the addition of maternal probiotics and synbiotics improved the piglet feed intake and altered the meat quality parameters, plasma metabolites, and gene expression related to meat quality.

## Introduction

Microbes colonizing the gut possess strong metabolic activity and play critical roles in regulating growth and development, nutrient metabolism, immune responses, and host health status (1). Previous studies have shown that the gut microbiota can affect muscle growth and fiber type transformation (2), thus influencing carcass traits and meat quality through multiple metabolic pathways. For example, dietary *Lactobacillus plantarum* supplementation can increase muscle mass in mice (3), whereas synbiotics supplementation in late-finishing pigs can enhance muscle antioxidant capacity and improve meat quality (4). Furthermore, dietary supplementation with the EM^®^Bokashi probiotic preparations containing specific *Lactobacillus* species and yeasts improved the slaughter value, macroelement (Mg, Ca, and Na) content, and chromatic color traits (b^*^ and C^*^) of meat (5). Multi-species probiotics supplementation also has beneficial effects on sensory evaluation, cooking yield, and drip loss of meat (6). Increasing evidence shows that maternal probiotics supplementation has beneficial effects on the growth and development of newborn piglets. For example, dietary *Bacillus* supplementation during late gestation and lactation has beneficial effects on body weight gain and intestinal microbiota modulation in piglets (7). In addition, dietary xylo-oligosaccharide (XOS) intervention may alter the proliferation of *Lactobacillus, Streptococcus*, and *Turicibacter*species, thus improving the growth performance of weaned piglets by regulating intestinal health (8). However, the mechanism by which the maternal gut microbiota regulates the growth performance and meat quality of offspring remains unknown.

Maternal health and nutritional status during gestation and lactation play a vital role in the meat quality of offspring. Several studies have demonstrated that maternal supplementation with functional additives during gestation and lactation can affect meat color, muscle tenderness, intramuscular fat (IMF), water-holding capacity, muscle fiber type, and muscle metabolism in offspring (9, 10). Furthermore, the fetal and neonatal periods are the most critical periods for skeletal muscle development (11). The development of postnatal muscle fibers also depends on the composition and types of fetal and neonatal muscle fibers (12). Therefore, maternal intervention during pregnancy and lactation is an effective strategy to improve the growth and development of skeletal muscle and meat quality of offspring.

Previous studies have demonstrated that supplementing *Bacillus altitudinis* during late gestation and lactation improves the feed efficiency of progeny in the early post-weaning period and increases the live weight at the end of the finishing period (13). In addition, dietary inclusion of *Saccharomyces cerevisiae* fermentation products from nursery to slaughter improved the marbling score of pork (14). Moreover, our previous studies showed that maternal addition of synbiotics (including *L. plantarum, S. cerevisiae*, and XOS) during pregnancy and lactation improved survival rate, nutrient metabolism, and intestinal barrier permeability, reduced oxidative stress, and modified colonic microbiota composition and metabolic activity in suckling piglets (15, 16). Bama mini-pigs, one of the most well-known native mini-pigs in China, have superior meat quality but slower muscle growth than commercial pigs (17). For local mini-pig breeds, different processing methods have different slaughter weight requirements. As reported by Cai et al. (18), 7.5–10-kg mini-pigs are generally used for roasting pork, whereas pigs with heavier body weights are used for processing bacons. Therefore, the present study used Bama mini-pig as a model to explore the effects of maternal addition of an antibiotic (inhibiting the gut microbiota), probiotics (increasing the beneficial gut microbiota), and synbiotics (increasing the beneficial gut microbiota and their fermenting substrate) during pregnancy and lactation on growth performance, carcass traits, meat quality, plasma metabolites, and related gene expression in offspring of different ages.

## Materials and Methods

### Animals, Diets, and Treatments

This study was conducted at the mini-pig experimental base in Goat Chong, Shimen town, Changde city, Hunan province, China. A total of 64 pregnant Bama mini-pigs with parities of 3–5 and initial body weight (BW) of 92.6 ± 11.76 kg were selected and randomly allocated into four groups (16 sows (pens) per group). The dietary treatment groups included the control (fed with an antibiotic-free basal diet), antibiotic (50 g/t virginiamycin with the basal diet), probiotics (200 mL/day probiotics mixture per animal with the basal diet), and synbiotics (500 g XOS per ton diet + 200 mL/day probiotics mixture per animal with the basal diet) groups. The probiotics mixture was provided by Hunan Lifeng Biotechnology Co., Ltd. (Changsha, Hunan, China) and contained *L. plantarum* ≥ 1 × 10^8^ CFU/mL and *S. cerevisiae* ≥0.2 × 10^8^ CFU/mL. XOS containing ≥ 35% xylobiose, xylotriose, and xylotetraose was provided by Shandong Longlive Biotechnology Co., Ltd. (Shandong, China). The supplemented probiotics mixture was mixed with the feed before feeding the sows, and XOS was added during feed production. The doses of probiotics and synbiotics were recommended by the manufacturers.

The sows were housed individually in gestation crates (2.2 m ×0.6 m) from days 1 to 105 of pregnancy, transferred to farrowing crates (2.2 m × 1.8 m) on day 106 of pregnancy, and remained there until piglets' weaning. The sows were fed with 0.8, 1, 1.2, 1.5, and 2 kg of pregnancy feed mix from days 1 to 5, 16–30, 31–75, 76–90, and 91–105 of pregnancy, respectively; they were fed with 1 kg of this feed mix a week before parturition and *ad libitum* after 3 days of parturition; then, they were fed with 2.4 kg of a lactation feed mix until weaning. The sows were fed twice daily at 8:00 am and 5:00 pm, and water was freely available at all times. Creep feed was provided to suckling piglets from 7 to 28 days of age. After weaning at 28 days of age, one male and one female piglet, close to the average BW per litter, were selected and transferred to the nursery house for the subsequent feeding trial. After 1 week of adaptation, four piglets from two litters in the same group were merged into one pen. There were eight pens (replicates) and 32 piglets per group. A total of 128 piglets were fed with the basal diet during the remaining days of the trial. The basal diet composition and nutrient levels of the sows and piglets are presented in Supplementary Tables 1, 2. Feeding and management were performed according to the standard operations of commercial pig farms.

### Determination of Growth Performance

The feed intake of the offspring per pen was recorded daily. The BW of 65-, 95-, and 125-day-old pigs was measured. Average daily gain (ADG), average daily feed intake (ADFI), and feed/gain (F/G) ratio were calculated for days 35–65, 66–95, and 96–125, respectively.

### Sample Collection

At 65, 95, and 125 days of age, the offspring from each group were fasted for 12 h and weighed, and then one pig per pen (eight pigs per group) was selected and euthanized under commercial conditions by electrical stunning (120 V, 200 Hz) and exsanguination. Blood samples were collected *via* the precaval vein in 10 mL heparinized tubes, centrifuged at 4°C and 3,500 × *g* for 10 min to obtain plasma, and immediately stored at −20°C for further analysis. After removing the head, legs, tail, and viscera, carcasses were split longitudinally to evaluate carcass traits and meat quality. *Longissimus thoracis* (LT) muscle samples were then collected and stored in sealed plastic bags at −20°C to analyze routine nutrients. LT muscle samples (~2 g) were also collected for mRNA analyses and were immediately frozen in liquid nitrogen and stored at −80°C.

### Measurement of Carcass Traits

According to Chinese pig raising industry standards (GB8467-87, 1988), the left side of each carcass was dissected into the skin, skeletal muscle, bone, and fat. The skeletal muscle, bone, fat, and leaf fat were weighed to calculate the percentage of live BW [tissue weight × 2 (kg)/BW (kg) × 100%] or the ratio to live BW [leaf fat weight × 2 (g)/BW (kg)]. Backfat thickness (between the sixth and seventh ribs) was measured using a Vernier caliper. The width and height of the LT muscle cross-section were measured using a Vernier caliper to calculate the loin-eye muscle area (width × height ×0.7).

### Assessment of Meat Quality

The subjective marbling and meat color scores of the LT muscle were determined following the National Pork Producers Council (NPPC) guidelines (19). Meat color, including a^*^ (redness), b^*^ (yellowness), and L^*^ (lightness) values of the LT muscle, was measured using a colorimeter (CR410; Konica Minolta Sensing, Inc., Tokyo, Japan) at 45 min post-mortem. The pH values at 45 min (pH_45min_) and 24 h (pH_24h_) post-mortem were measured using a portable pH meter (Russell CD700; Russell pH Limited, Germany). Drip loss was determined using the “bag method” as previously described by Honikel (20). The LT muscle samples stored at 4°C were placed in individual polyethylene vacuum bags and cooked for 30 min, and an internal temperature of 70°C was detected with a thermocouple thermometer (Digital Thermometer TP101; Changzhou KB Instruments & Meter Co., Ltd., Changzhou, China) in a water bath at 75°C for 24 h post-mortem (21). The cooked samples were cooled to room temperature, dried with a paper towel, and re-weighed to calculate the cooking yield [(cooked weight/fresh weight) × 100%]. After the cooking yield was determined, the samples were trimmed parallel to the muscle fiber into strips with a diameter of 12.7 mm and a length of 20 mm. The stripes were then used to determine shear force (N) using a texture analyzer (FTC-TMS/PRO; FTC Corporation, Virginia, United States) with a load cell of 15 kg and a crosshead speed of 200 mm/min.

### Chemical Composition Analysis of LT Muscle

The LT muscle samples were minced after weighing and dried in a vacuum-freeze dryer at 20 ± 5 Pa and −45 ± 5°C for 72 h (CHRIST RVC2-25 CDPIUS; Christ Company, Oster ode, Germany) to calculate dry matter (DM) content. Crude protein (CP) content (N × 6.25) was determined using the Kjeldahl method and following the standards provided by the Association of Official Analytical Chemists (AOAC) (22). IMF content was determined using the Soxhlet extraction method (23).

Approximately 0.1 g of freeze-dried skeletal muscle powder was hydrolyzed in 10 mL of a 6 mol/L HCl solution in a sealed ampoule bottle at 110°C for 24 h. The suspension was diluted to 100 mL using double-distilled water in a volumetric flask (24), and 1 mL of the supernatant was transferred to 1.5 mL centrifuge tubes and evaporated to dryness in a water bath at 65°C. The samples were then dissolved using 1 mL of 0.02 mol/L HCl solution and filtered through a 0.45-μm membrane prior to analysis of hydrolyzed amino acid (AA) content using an automatic amino acid analyzer (L-8900; Hitachi, Tokyo, Japan) (25).

Medium- and long-chain fatty acids (FAs) were identified by gas-liquid chromatography (7890A; Agilent, CA, United States). Briefly, the FAs were analyzed using an FID detector after extraction with a mixture of benzene and petroleum ether and by methyl esterification using potassium hydroxide methanol. The sample pretreatment method used was performed as described previously by Liu et al. (26). The GC conditions were as follows: the injector and detector temperatures were at 280°C; the initial column temperature was at 100°C for 13 min, increased at 10°C/min to 180°C, for 6 min, increased at 1°C/min to 200°C, for 20 min, then increased at 4°C/min to 230°C, for 15 min. Nitrogen was used as a carrier gas at a flow rate of 0.8 mL/min. The inlet temperature was 270°C, injection volume was 1 μL, and split ratio was 20:1. FA content was determined by comparison with retention times of the reference standard mixtures (99%, Sigma, St. Louis, MO, United States). Data were expressed as a percentage of total FAs.

### Muscular RNA Extraction and Gene Expression Analysis

The total RNA of the LT muscle was extracted using the AG RNAex Pro reagent (Accurate Biology, Hunan, China) according to the manufacturer's protocol. The concentration of the extracted RNA was measured using a NanoDrop ND-2000 spectrophotometer (Thermo Fisher Scientific, Waltham, MA, United States), and purity was determined using the A260/A280 ratio. RNA quality was evaluated by agarose gel electrophoresis. The total RNA (1,000 ng) was reverse-transcribed into cDNA using the Evo M-MLV RT Kit with gDNA clean for quantitative PCR analysis (Accurate Biology). An RT-PCR analysis was performed on the LightCycler^®^ 480II Real-Time PCR System (Roche, Basel, Switzerland) with SYBR^®^ Green Premix Pro Taq HS qPCR Kit (Accurate Biology). Specific primers for the target genes were designed and synthesized by Sangon Biotech Co., Ltd. (Shanghai, China) (Supplementary Table 3). RT-qPCR was performed in a 10-μL reaction system, including 0.25 μL of each primer, 5 μL SYBR^®^ Green Premix, 2 μL cDNA, and 2.5 μL double-distilled water. The PCR cycling conditions were as per the instructions for the SYBR Green Premix. Relative gene expression levels between the different groups were calculated using the 2^−ΔΔ*Ct*^ method (27).

### Determination of Plasma Biochemical Parameters

The plasma levels of alanine aminotransferase (ALT), alkaline phosphatase (ALP), α-amylase (α-AMS), aspartate aminotransferase (AST), cholinesterase (CHE), lactate dehydrogenase (LDH), albumin (ALB), ammonia (AMM), total protein (TP), urea nitrogen (UN), high-density lipoprotein cholesterol (HDL-C), low-density lipoprotein cholesterol (LDL-C), total cholesterol (TC), triglyceride (TG), and glucose (GLU) were determined using commercially available kits (F. Hoffmann-La Roche Ltd., Basel, Switzerland) and a Roche automatic biochemical analyzer (Cobas c311; F. Hoffmann-La Roche Ltd., Basel, Switzerland).

### Determination of Plasma Free Amino Acids

The plasma samples (800 μL) were mixed with 8% sulfosalicylic acid in equal proportions and then stored at 4°C overnight to precipitate proteins. Supernatants were obtained by centrifugation at 8,000 × *g* for 10 min. The processed samples were then filtered through a 0.22-μm membrane into sample bottles prior to analysis of free AAs using an automatic amino acid analyzer (L8900; Hitachi, Tokyo, Japan).

### Statistical Analysis

The experimental unit was a pen for ADFI, ADG, and F/G data, and individual pigs were used for data on carcass traits, meat quality, muscular chemical composition, plasma biochemical parameters, plasma-free AAs, and mRNA expression. All the data were analyzed using the SPSS software package (SPSS v. 25.0; SPSS Inc., Chicago IL, United States). The normal distribution of the data was confirmed by the Shapiro-Wilk test before assessing differences between the four groups. The data were analyzed by one-way ANOVA, and differences between the four treatments were compared by Tukey's *post-hoc* test. The data are presented as mean ± SEM. The differences were considered statistically significant at *P* < 0.05.

## Results

### Growth Performance

The effects of probiotics and synbiotics addition in sows' diets on growth performance of the offspring are shown in Table 1. Compared with the antibiotic group, the addition of maternal probiotics increased (*P* < 0.05) the BW of 95-day-old pigs. The ADFI increased in the probiotics group and decreased in the antibiotic group at 66–95 days of age when compared with the control group (*P* < 0.05), and the antibiotic group had higher F/G than the control group and higher ADFI than the other three groups at 96–125 days of age (*P* < 0.05).

**Table 1:** Effects of probiotics and synbiotics addition in sows' diets on growth performance of offspring pigs.

**Items**	**Control**	**Antibiotic**	**Probiotics**	**Synbiotics**	**SEM**	***P-*values**
	**group**	**group**	**group**	**group**		
**Body weight (kg)**						
35 days of age	4.97	4.90	4.80	4.66	0.172	0.609
65 days of age	9.37	8.99	9.54	9.26	0.190	0.243
95 days of age	14.05^ab^	12.37^b^	14.61^a^	13.32^ab^	0.528	0.032
125 days of age	22.67	26.19	25.36	27.19	1.289	0.097
**Average daily gain (kg/d)**						
35–65 days of age	0.15	0.14	0.15	0.16	0.005	0.079
66–95 days of age	0.18	0.15	0.18	0.16	0.012	0.284
96–125 days of age	0.27	0.27	0.25	0.26	0.027	0.902
**Average daily feed intake (kg/d)**						
35–65 days of age	0.41	0.41	0.44	0.41	0.012	0.330
66–95 days of age	0.64^b^	0.55^c^	0.75^a^	0.70^ab^	0.024	<0.0001
96–125 days of age	0.92^b^	1.32^a^	0.91^b^	1.10^b^	0.065	<0.0001
**Feed/gain ratio**						
35–65 days of age	3.02	2.86	2.68	2.78	0.102	0.136
66–95 days of age	3.56	3.82	3.75	4.25	0.220	0.173
96–125 days of age	3.32^b^	4.17^a^	4.00^ab^	3.90^ab^	0.211	0.040

*Data are presented as means with pooled SEM. Values in the same row with different superscript letters were significantly different (P < 0.05). The replicates per group at 35–65 and 66–95 days of age were 8; at 96–125 days of age, the replicates of the control group, antibiotic group, probiotics group, and synbiotics group were 8, 6, 8, and 6, respectively*.

### Carcass Traits

The effects of maternal probiotics and synbiotics addition on the carcass traits of the offspring are presented in Table 2. At 65 days of age, the probiotics group had a higher (*P* < 0.05) carcass weight than the antibiotic and synbiotics groups, whereas the backfat thickness was higher (*P* < 0.05) in the probiotics group than in the control and antibiotic groups. At 95 days of age, the leaf fat ratio was lower (*P* < 0.05) in the antibiotic and probiotics groups than in the control and synbiotics groups, while the loin-eye area was higher (*P* < 0.05) in the synbiotics group than in the antibiotic group. Moreover, the antibiotic group had a lower fat percentage than the other three groups and a lower carcass weight than the control group at 95 days of age (*P* < 0.05). At 125 days of age, the addition of maternal antibiotic, probiotics, and synbiotics increased the fat percentage and backfat thickness compared with those in the control group, whereas the addition of maternal synbiotics decreased the muscle percentage of offspring pigs compared to that in the control and probiotics groups (*P* < 0.05). The addition of maternal synbiotics decreased (*P* < 0.05) the loin-eye area of offspring pigs compared to that in the other three groups at 125 days of age.

**Table 2:** Effects of probiotics and synbiotics addition in sows' diets on carcass traits of offspring pigs.

**Items**	**Control**	**Antibiotic**	**Probiotics**	**Synbiotics**	**SEM**	***P-*values**
	**group**	**group**	**group**	**group**		
**Carcass weight (kg)**						
65 days of age	5.00^ab^	4.71^b^	5.27^a^	4.82^b^	0.108	0.006
95 days of age	8.25^a^	6.36^b^	7.73^ab^	7.32^ab^	0.445	0.037
125 days of age	13.83	16.40	14.60	16.25	0.817	0.091
**Muscle percentage (%)**						
65 days of age	21.56	21.09	22.49	21.17	0.491	0.191
95 days of age	21.23	20.67	22.00	21.51	0.389	0.130
125 days of age	23.87^a^	23.11^ab^	23.53^a^	22.44^b^	0.236	0.002
**Fat percentage (%)**						
65 days of age	12.14	12.10	13.31	11.78	0.396	0.052
95 days of age	15.24^a^	12.47^b^	15.29^a^	15.01^a^	0.662	0.011
125 days of age	17.73^b^	21.49^a^	22.28^a^	20.89^a^	0.548	<0.0001
**Bone percentage (%)**						
65 days of age	11.80	11.89	11.95	11.98	0.190	0.915
95 days of age	10.13	10.44	10.57	9.99	0.273	0.424
125 days of age	9.15	8.55	9.60	9.25	0.261	0.069
**Leaf fat ratio (g/kg)**						
65 days of age	6.93	6.04	7.05	6.75	0.376	0.248
95 days of age	11.41^a^	8.09^b^	7.77^b^	11.17^a^	0.587	<0.0001
125 days of age	16.77^b^	17.88^b^	21.54^a^	19.33^ab^	0.925	0.005
**Backfat thickness (mm)**						
65 days of age	13.27^b^	12.78^b^	14.65^a^	13.90^ab^	0.396	0.014
95 days of age	18.79	16.78	18.46	16.98	0.611	0.060
125 days of age	24.75^b^	28.81^a^	27.53^a^	28.15^a^	0.832	0.009
**Loin-eye muscle area (cm** ^**2**^ **)**						
65 days of age	4.21	4.29	4.45	4.19	0.200	0.788
95 days of age	4.33^ab^	4.22^b^	4.39^ab^	5.01^a^	0.200	0.038
125 days of age	7.20^a^	7.36^a^	7.67^a^	5.57^b^	0.282	<0.0001

*Data are presented as means with pooled SEM. Values in the same row with different superscript letters were significantly different (P < 0.05). The replicates per group at 65 and 95 days of age were 8; at 125 days of age, the replicates of the control group, antibiotic group, probiotics group, and synbiotics group were 8, 6, 8, and 6, respectively*.

### Meat Quality

The effects of adding dietary probiotics and synbiotics to sows' diets on the meat quality of offspring are shown in Table 3. Drip loss in the probiotics and synbiotics groups was lower (*P* < 0.05) than that in the control and antibiotic groups, whereas cooking yield in the probiotics group was higher (*P* < 0.05) at 65 days of age. In addition, the L^*^ value was lower (*P* < 0.05) in the probiotics group, and shear force was higher (*P* < 0.05) in the synbiotics group than in the other three groups at 65 days of age. At 95 days of age, the a^*^ value was lower (*P* < 0.05) in the probiotics group but higher (*P* < 0.05) in the synbiotics group than in the control and antibiotic groups. pH_45min_ values was higher (*P* < 0.05) in the probiotics group than in the control and synbiotics groups, and the L^*^ value in the probiotics group was higher (*P* < 0.05) than in the synbiotics group at 95 days of age. At 125 days of age, the L^*^ value was lower in the synbiotics group than in the control and probiotics groups, whereas the b^*^ value was higher in the probiotics group than in the antibiotic and synbiotics groups (*P* < 0.05). The shear force in the probiotics and synbiotics groups was lower than in the control and antibiotic groups, while cooking yield and shear force were higher in the antibiotic group than in the other three groups at 125 days of age (*P* < 0.05).

**Table 3:** Effects of probiotics and synbiotics addition in sows' diets on meat quality of offspring pigs.

**Items**	**Control**	**Antibiotic**	**Probiotics**	**Synbiotics**	**SEM**	***P-*values**
	**group**	**group**	**group**	**group**		
**Marbling score**						
65 days of age	1.75	1.63	1.38	1.50	0.180	0.502
95 days of age	1.88	1.38	1.25	1.38	0.190	0.120
125 days of age	1.50	1.67	1.25	1.67	0.193	0.376
**Meat color score**						
65 days of age	3.13	2.88	2.75	2.50	0.336	0.620
95 days of age	2.50	2.38	2.38	2.63	0.184	0.740
125 days of age	2.75	3.17	2.50	2.83	0.208	0.188
a*value						
65 days of age	19.58	19.11	19.47	18.50	0.617	0.605
95 days of age	18.44^b^	18.01^b^	17.12^c^	19.86^a^	0.226	<0.0001
125 days of age	18.14	17.89	17.17	17.16	0.499	0.377
b*value						
65 days of age	6.87	6.82	7.50	7.05	0.192	0.071
95 days of age	6.72	6.59	6.06	6.47	0.213	0.171
125 days of age	7.07^ab^	6.50^b^	7.30^a^	6.48^b^	0.187	0.008
L*value						
65 days of age	51.01^a^	50.66^a^	48.11^b^	50.75^a^	0.623	0.008
95 days of age	50.16^ab^	49.67^ab^	51.78^a^	48.50^b^	0.766	0.041
125 days of age	50.94^a^	49.49^ab^	52.35^a^	47.33^b^	0.993	0.011
**pH**_**45min**_						
65 days of age	6.55	6.55	6.50	6.52	0.035	0.744
95 days of age	6.54^b^	6.67^ab^	6.74^a^	6.55^b^	0.037	0.002
125 days of age	6.49	6.63	6.66	6.52	0.054	0.069
**pH**_**24h**_						
65 days of age	5.46	5.49	5.61	5.45	0.053	0.134
95 days of age	5.46	5.44	5.46	5.49	0.013	0.088
125 days of age	5.46	5.53	5.50	5.50	0.025	0.245
**Drip loss (%)**						
65 days of age	6.32^a^	5.64^a^	4.41^b^	2.96^c^	0.340	<0.0001
95 days of age	3.42	3.35	2.98	2.78	0.313	0.437
125 days of age	4.82	3.31	3.82	3.94	0.485	0.182
**Cooking yield (%)**						
65 days of age	63.01^bc^	60.94^c^	67.02^a^	65.22^ab^	1.087	0.003
95 days of age	70.92	69.26	70.99	68.53	0.806	0.098
125 days of age	68.57^b^	78.41^a^	68.50^b^	66.94^b^	2.331	0.012
**Shear force (N)**						
65 days of age	61.50^b^	62.96^b^	58.73^b^	75.28^a^	2.311	<0.0001
95 days of age	73.25	64.61	65.74	70.82	2.936	0.143
125 days of age	91.94^b^	101.74^a^	77.14^c^	84.41^c^	2.516	<0.0001

*Data are presented as means with pooled SEM. Values in the same row with different superscript letters were significantly different (P < 0.05). The replicates per group at 65 and 95 days of age were 8; at 125 days of age, the replicates of the control group, antibiotic group, probiotics group, and synbiotics group were 8, 6, 8, and 6, respectively*.

a^*^, redness;

b^*^, yellowness;

L^*^, lightness.

#### Amino Acid Content of LT Muscle

The effects of maternal probiotics and synbiotics addition on AA content in the LT muscle are presented in Table 4. The asparagine (Asp) and glycine (Gly) contents in the LT muscle were lower in the probiotics group than in the control and antibiotic groups at 65 days of age (*P* < 0.05). In addition, the histidine (His) content in the LT muscle was higher (*P* < 0.05) in the probiotics group than in the other three groups at 65 days of age.

**Table 4:** Effects of probiotics and synbiotics addition in sows' diets on crude protein and amino acid contents in the *longissimus thoracis* muscle of offspring pigs (g/100 g fresh muscle).

**Items**	**Control**	**Antibiotic**	**Probiotics**	**Synbiotics**	**SEM**	***P-*values**
	**group**	**group**	**group**	**group**		
**CP**						
65 days of age	23.39^ab^	23.80^ab^	24.50^a^	23.17^b^	0.318	0.032
95 days of age	22.12	22.65	22.83	22.26	0.316	0.365
125 days of age	22.21^ab^	22.79^a^	22.06^ab^	21.44^b^	0.252	0.016
**Ala**						
65 days of age	1.20	1.20	1.18	1.20	0.031	0.975
95 days of age	1.06	1.14	1.12	1.07	0.031	0.237
125 days of age	1.17	1.23	1.27	1.21	0.029	0.126
**Arg**						
65 days of age	1.34	1.31	1.27	1.31	0.026	0.378
95 days of age	1.21^b^	1.30^a^	1.35^a^	1.34^a^	0.032	0.017
125 days of age	1.34	1.37	1.43	1.38	0.031	0.256
**Asp**						
65 days of age	1.80^a^	1.80^a^	1.63^b^	1.70^ab^	0.038	0.009
95 days of age	1.51^b^	1.55^ab^	1.68^a^	1.69^a^	0.044	0.014
125 days of age	1.76^b^	1.91^a^	1.94^a^	1.77^b^	0.041	0.004
**Glu**						
65 days of age	2.63	2.63	2.53	2.54	0.070	0.632
95 days of age	2.63	2.68	2.82	2.81	0.066	0.144
125 days of age	2.84	2.96	3.03	3.00	0.070	0.215
**Gly**						
65 days of age	1.09^a^	1.08^a^	0.96^b^	1.01^ab^	0.034	0.047
95 days of age	0.95	1.01	1.05	1.06	0.035	0.118
125 days of age	0.98	0.96	1.01	0.96	0.040	0.817
**His**						
65 days of age	0.80^b^	0.75^b^	0.91^a^	0.79^b^	0.021	<0.0001
95 days of age	0.94^a^	0.90^ab^	0.82^b^	0.85^ab^	0.027	0.022
125 days of age	0.89^b^	0.95^b^	0.94^b^	1.08^a^	0.027	<0.0001
**Ile**						
65 days of age	0.90	0.90	0.83	0.87	0.021	0.056
95 days of age	0.82^b^	0.83^b^	0.91^a^	0.91^a^	0.021	0.002
125 days of age	0.99	1.04	1.06	0.98	0.024	0.075
**Leu**						
65 days of age	1.74	1.71	1.61	1.68	0.034	0.079
95 days of age	1.43	1.42	1.50	1.51	0.037	0.252
125 days of age	1.67^b^	1.80^a^	1.82^a^	1.71^ab^	0.040	0.033
**Lys**						
65 days of age	1.77	1.77	1.64	1.73	0.040	0.105
95 days of age	1.59^b^	1.58^b^	1.72^ab^	1.78^a^	0.047	0.014
125 days of age	1.89	1.96	2.02	1.91	0.045	0.194
**Met**						
65 days of age	0.53	0.54	0.50	0.49	0.016	0.278
95 days of age	0.54	0.52	0.54	0.53	0.013	0.612
125 days of age	0.62	0.65	0.66	0.66	0.019	0.362
**Phe**						
65 days of age	0.87	0.84	0.86	0.87	0.021	0.789
95 days of age	0.86	0.81	0.84	0.87	0.020	0.193
125 days of age	0.90^b^	0.91^ab^	0.96^ab^	1.00^a^	0.025	0.039
**Pro**						
65 days of age	0.94	0.96	1.07	0.96	0.039	0.094
95 days of age	1.17^ab^	1.33^a^	1.14^ab^	0.98^b^	0.061	0.006
125 days of age	1.04	0.90	0.97	1.07	0.050	0.136
**Ser**						
65 days of age	0.69	0.71	0.67	0.68	0.020	0.518
95 days of age	0.71	0.74	0.76	0.75	0.017	0.172
125 days of age	0.75^b^	0.75^b^	0.78^b^	0.84^a^	0.019	0.012
**Tyr**						
65 days of age	0.71	0.67	0.70	0.70	0.016	0.341
95 days of age	0.63	0.70	0.71	0.70	0.038	0.428
125 days of age	0.75	0.70	0.75	0.80	0.026	0.176
**Thr**						
65 days of age	0.93	0.93	0.87	0.90	0.018	0.051
95 days of age	0.84	0.88	0.92	0.89	0.024	0.120
125 days of age	0.96	0.97	1.00	0.99	0.024	0.493
**Val**						
65 days of age	0.97	0.97	0.90	0.95	0.023	0.107
95 days of age	0.96	0.93	0.98	1.00	0.023	0.130
125 days of age	1.08	1.11	1.14	1.12	0.027	0.447
**EAA**						
65 days of age	9.83	9.72	9.49	9.59	0.195	0.637
95 days of age	9.17	9.18	9.58	9.68	0.216	0.233
125 days of age	10.35	10.76	11.03	10.84	0.234	0.190
**NEAA**						
65 days of age	9.08	9.06	8.76	8.80	0.214	0.610
95 days of age	8.56	9.14	9.28	9.06	0.229	0.159
125 days of age	9.29	9.42	9.73	9.64	0.226	0.469
**FAA**						
65 days of age	8.06	8.03	7.57	7.76	0.181	0.211
95 days of age	7.39	7.68	8.01	7.97	0.180	0.075
125 days of age	8.09	8.43	8.66	8.31	0.188	0.176
**TAA**						
65 days of age	18.90	18.78	18.27	18.39	0.402	0.643
95 days of age	17.25^b^	18.32^ab^	18.85^a^	18.74^a^	0.403	0.035
125 days of age	19.64	20.18	20.76	20.4	0.451	0.303

*Data are presented as means with pooled SEM. Values in the same row with different superscript letters were significantly different (P < 0.05). The replicates per group at 65 and 95 days of age were 8; at 125 days of age, the replicates of the control group, antibiotic group, probiotics group, and synbiotics group were 8, 6, 8, and 6, respectively. CP, crude protein; EAA, essential amino acid; NEAA, nonessential amino acid; FAA, flavor amino acid; TAA, total amino acid. The EAAs included arginine (Arg), histidine (His), isoleucine (Ile), leucine (Leu), lysine (Lys), methionine (Met), phenylalanine (Phe), threonine (Thr), and valine (Val). The NEAAs included alanine (Ala), aspartate (Asp), glutamate (Glu), glycine (Gly), proline (Pro), serine (Ser), and tyrosine (Tyr). The FAAs included Asp, Glu, Gly, Ala, and Arg*.

At 95 days of age, the arginine (Arg) content in the LT muscle was higher (*P* < 0.05) in the antibiotic, probiotics, and synbiotics groups than in the control group. Compared to the control group, the Asp and total AA (TAA) contents in the LT muscle were higher in the probiotics and synbiotics groups, whereas His content was lower in the probiotics group (*P* < 0.05). The isoleucine (Ile) content in the probiotics and synbiotics groups and the lysine (Lys) content in the synbiotics group were higher than those in the control and antibiotic groups (*P* < 0.05). The proline (Pro) content was lower in the synbiotics group than in the antibiotic group (*P* < 0.05).

At 125 days of age, the leucine (Leu) content in the LT muscle of the antibiotic and probiotics groups and the phenylalanine (Phe) content in the synbiotics group were higher than in the control group, whereas Asp content was higher in the antibiotic and probiotics groups than in the control and synbiotics groups (*P* < 0.05), and the serine (Ser) and His contents in the LT muscle were higher (*P* < 0.05) in the synbiotics group than in the other three groups at 125 days of age.

#### Fatty Acid Content of LT Muscle

The effects of maternal probiotics and synbiotics addition on FA content in the LT muscle of the offspring are shown in Table 5. At 65 days of age, the LT muscle C18:2n6c and polyunsaturated FA (PUFA) contents in the antibiotic and probiotics groups and the C16:0 content in the probiotics group were higher than those in the control and synbiotics groups, whereas C12:0 content was higher in the synbiotics group than in the control group, and the C14:0 content in the synbiotics group was higher than that in the control and probiotics groups (*P* < 0.05). In addition, C24:0 content was higher (*P* < 0.05) in the probiotics group, while C18:1n9c, C20:0, C20:1, and SFA saturated FA (SFA) contents were lower (*P* < 0.05) compared with the other three groups. C10:0 content was higher (*P* < 0.05) in the synbiotics group than in the other three groups, and IMF content was lower (*P* < 0.05) in the probiotics group than in the antibiotic and synbiotics groups.

**Table 5:** Effects of probiotics and synbiotics addition in sows' diets on intramuscular fat and fatty acid contents in the *longissimus thoracis* muscle of offspring pigs (%).

**Items**	**Control**	**Antibiotic**	**Probiotics**	**Synbiotics**	**SEM**	***P-*values**
	**group**	**group**	**group**	**group**		
**Intramuscular fat**						
65 days of age	2.72^ab^	3.13^a^	2.32^b^	3.24^a^	0.231	0.035
95 days of age	2.62^ab^	2.44^ab^	3.21^a^	1.52^b^	0.325	0.009
125 days of age	2.15^b^	2.07^b^	1.79^b^	2.80^a^	0.197	0.012
**C10:0**						
65 days of age	0.024^b^	0.023^b^	0.021^b^	0.028^a^	0.001	0.004
95 days of age	0.022^b^	0.024^ab^	0.027^a^	0.026^a^	0.001	0.028
125 days of age	0.024	0.023	0.026	0.029	0.002	0.145
**C12:0**						
65 days of age	0.036^b^	0.039^ab^	0.038^ab^	0.042^a^	0.001	0.039
95 days of age	0.041^a^	0.030^b^	0.035^b^	0.031^b^	0.002	<0.0001
125 days of age	0.028^a^	0.023^b^	0.028^a^	0.029^a^	0.001	0.024
**C14:0**						
65 days of age	0.447^bc^	0.486^ab^	0.423^c^	0.524^a^	0.014	<0.0001
95 days of age	0.487^b^	0.514^ab^	0.559^a^	0.536^ab^	0.017	0.040
125 days of age	0.497	0.505	0.474	0.509	0.027	0.772
**C15:0**						
65 days of age	0.024	0.029	0.026	0.027	0.001	0.152
95 days of age	0.022	0.024	0.023	0.026	0.001	0.156
125 days of age	0.022^a^	0.019^ab^	0.017^b^	0.016^b^	0.001	0.001
**C16:0**						
65 days of age	8.137^a^	8.176^ab^	7.604^b^	8.355^a^	0.162	0.017
95 days of age	8.334	8.209	8.643	8.301	0.141	0.172
125 days of age	8.231	8.290	7.796	8.089	0.216	0.356
**C16:1**						
65 days of age	1.177	1.169	1.091	1.254	0.055	0.253
95 days of age	0.728^c^	0.906^ab^	0.976^a^	0.884^b^	0.025	<0.0001
125 days of age	0.785^b^	0.755^b^	0.868^a^	0.757^b^	0.026	0.013
**C17:0**						
65 days of age	0.103	0.116	0.100	0.110	0.005	0.183
95 days of age	0.091	0.094	0.094	0.100	0.004	0.514
125 days of age	0.082^a^	0.078^ab^	0.061^b^	0.070^ab^	0.006	0.045
**C18:0**						
65 days of age	4.364	4.207	4.062	4.244	0.085	0.118
95 days of age	5.141	4.824	5.001	4.759	0.103	0.056
125 days of age	4.670	4.440	4.687	4.621	0.102	0.345
**C18:1n9c**						
65 days of age	8.753^a^	8.813^a^	8.023^b^	8.805^a^	0.198	0.021
95 days of age	8.820^ab^	8.592^b^	9.663^a^	8.894^ab^	0.256	0.035
125 days of age	9.722	9.520	9.567	10.171	0.358	0.610
**C18:1n9t**						
65 days of age	0.036	0.036	0.035	0.033	0.001	0.500
95 days of age	0.034	0.032	0.035	0.032	0.002	0.538
125 days of age	0.042^a^	0.035^b^	0.035^b^	0.036^b^	0.002	0.019
**C18:2n6c**						
65 days of age	4.135^b^	4.499^a^	4.626^a^	4.008^b^	0.107	0.001
95 days of age	3.179	3.503	3.242	3.165	0.104	0.101
125 days of age	2.603^a^	2.306^ab^	2.405^ab^	2.150^b^	0.105	0.037
**C20:0**						
65 days of age	0.063^a^	0.064^a^	0.051^b^	0.068^a^	0.003	0.001
95 days of age	0.070	0.074	0.075	0.068	0.003	0.190
125 days of age	0.063	0.057	0.061	0.064	0.003	0.387
**C20:1**						
65 days of age	0.204^b^	0.223^ab^	0.181^c^	0.241^a^	0.008	<0.0001
95 days of age	0.303^a^	0.223^c^	0.296^a^	0.263^b^	0.010	<0.0001
125 days of age	0.097	0.088	0.090	0.085	0.003	0.139
**C20:2**						
65 days of age	0.163	0.155	0.143	0.156	0.005	0.059
95 days of age	0.153	0.138	0.148	0.138	0.006	0.267
125 days of age	0.105	0.091	0.091	0.088	0.005	0.097
**C20:3n6**						
65 days of age	0.101	0.108	0.114	0.116	0.005	0.241
95 days of age	0.080	0.090	0.073	0.087	0.006	0.146
125 days of age	0.072	0.073	0.078	0.068	0.006	0.700
**C20:4n6**						
65 days of age	0.858	0.841	0.936	0.790	0.069	0.522
95 days of age	0.512^b^	0.762^a^	0.499^b^	0.614^b^	0.049	0.002
125 days of age	0.521	0.562	0.588	0.448	0.051	0.274
**C24:0**						
65 days of age	0.052^b^	0.056^b^	0.074^a^	0.054^b^	0.004	0.002
95 days of age	0.051^a^	0.055^a^	0.036^b^	0.046^ab^	0.004	0.010
125 days of age	0.048	0.054	0.057	0.057	0.004	0.401
**SFA**						
65 days of age	13.267^a^	13.245^a^	12.506^b^	13.271^a^	0.219	0.049
95 days of age	14.218	13.855	14.391	13.757	0.234	0.204
125 days of age	13.712	12.881	13.271	13.111	0.393	0.496
**UFA**						
65 days of age	15.777	15.921	15.444	15.608	0.226	0.482
95 days of age	14.318^b^	14.175^b^	15.186^a^	14.553^b^	0.161	0.001
125 days of age	14.061	13.697	13.528	13.803	0.302	0.604
**MUFA**						
65 days of age	10.144	10.901	9.373	10.512	0.447	0.119
95 days of age	9.857^b^	9.568^b^	10.94^a^	10.084^b^	0.267	0.007
125 days of age	10.761	10.465	10.475	10.87	0.405	0.863
**PUFA**						
65 days of age	5.404^b^	6.389^a^	6.400^a^	5.343^b^	0.314	0.027
95 days of age	3.891^b^	4.587^a^	4.094^ab^	4.198^ab^	0.156	0.028
125 days of age	3.300	3.232	3.192	2.933	0.215	0.674
**PUFA/SFA**						
65 days of age	0.408	1.164	0.485	0.409	0.311	0.267
95 days of age	0.276	0.328	0.283	0.307	0.015	0.082
125 days of age	0.246	0.244	0.241	0.226	0.020	0.913

*Data are presented as means with pooled SEM. Values in the same row with different superscript letters were significantly different (P < 0.05). The replicates per group at 65 and 95 days of age were 8; at 125 days of age, the replicates of the control group, antibiotic group, probiotics group, and synbiotics group were 8, 6, 8, and 6, respectively. SFA, saturated fatty acid; UFA, unsaturated fatty acid; MUFA, monounsaturated fatty acid; PUFA, polyunsaturated fatty acid. The SFAs include C10:0, C12:0, C14:0, C15:0, C16:0,C17:0, C18:0, C20:0, and C24:0; the UFAs include C16:1, C18:1n9c, C18:1n9t, C18:2n6c, C18:3n3, C20:1, C20:2, C20:3n6, and C20:4n6; the MUFAs include C16:1, C18:1n9c, C18:1n9t, and C20:1; the PUFAs include C18:2n6c, C18:3n3, C20:2, C20:3n6, and C20:4n6*.

At 95 days of age, the C10:0 content in the probiotics and synbiotics groups and the C14:0 content in the probiotics group was higher, while the C20:1 content in the antibiotic and synbiotics groups was lower than in the control group (*P* < 0.05). In addition, C16:1 content was higher, while C12:0 content was lower in the antibiotic, probiotics, and synbiotics groups than in the control group (*P* < 0.05). Moreover, the unsaturated FA (UFA) and monounsaturated FA (MUFA) contents were higher (*P* < 0.05) in the probiotics group than in the other three groups at 95 days of age.

At 125 days of age, the C15:0 content in the LT muscle of the probiotics and synbiotics groups, C17:0 content in the probiotics group, C18:2n6c content in the synbiotics group, and C18:1n9t content in the antibiotic, probiotics, and synbiotics groups were lower (*P* < 0.05) than in the control group. In addition, the C16:1 content in the probiotics group and the IMF in the synbiotics group were higher (*P* < 0.05) than those in the other three groups.

#### The mRNA Expression of Genes in LT Muscle

**Figure 1:**
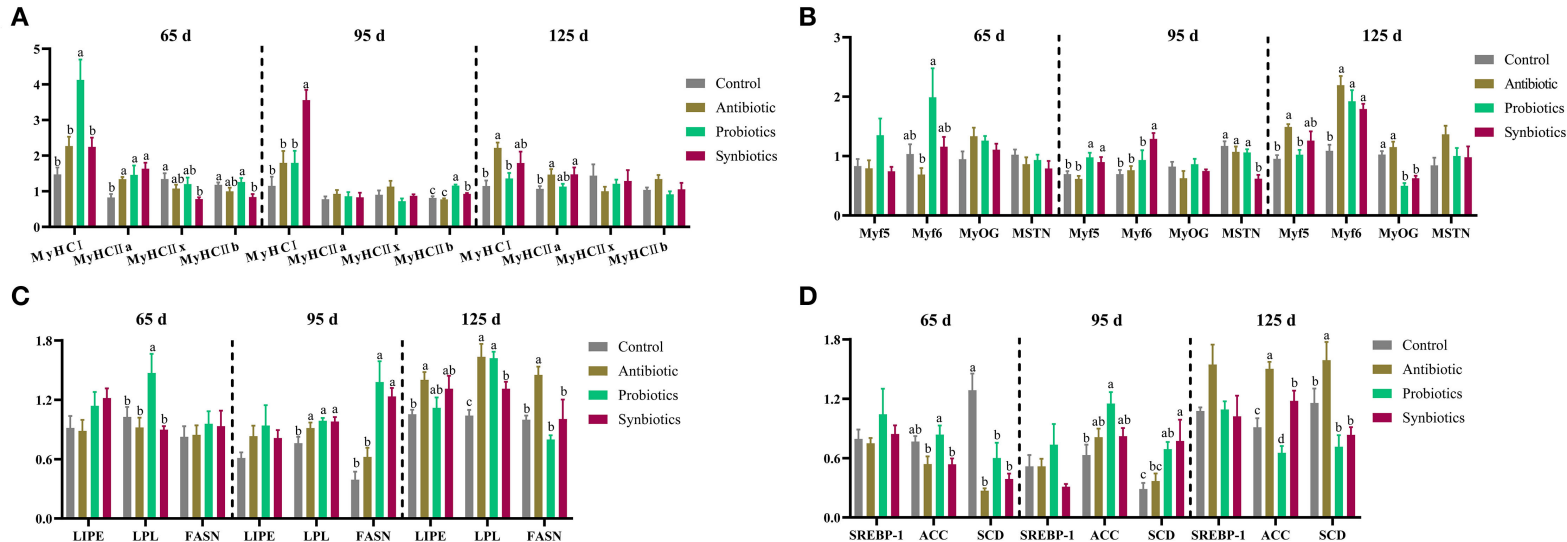
Effects of probiotics and synbiotics addition in sows' diets on **(A)** mRNA expression of myosin heavy chain (MyHC) isoforms, **(B)** myogenic regulatory factors (MRFs), and **(C,D)** lipid metabolism-related genes in the *longissimus thoracis* muscle of offspring pigs at 65, 95, and 125 days of age. 65 d, 65 days of age; 95 d, 95 days of age; 125 d, 125 days of age. *ACC*, acetyl-CoA carboxylase; *FASN*, fatty acid synthase; *LIPE*, hormone-sensitive triglyceride lipase; *LPL*, lipoprotein lipase; *Myf5*, myogenic factor 5; *Myf6*, myogenic factor 6; *MyOG*, myogenin; *MSTN*, myostatin; *SCD*, stearoyl-CoA desaturase; *SREBP-1*, sterol-regulatory element-binding protein-1.

The effects of probiotics and synbiotics addition in sows' diets on the mRNA expression of myosin heavy chain (MyHC) isoforms, myogenic regulatory factors (MRFs), and lipid metabolism-related genes in the LT muscle of the offspring are shown in Figure 1. Compared with the control group, the mRNA expression of *MyHC IIa* in the antibiotic, probiotics, and synbiotics groups were upregulated (*P* < 0.05), whereas that of *MyHC IIx* in the synbiotics group was downregulated (*P* < 0.05) at 65 days of age (Figures 1A,B). The mRNA expression of *MyHC IIb* in the synbiotics group was downregulated compared with that in the control and probiotics groups, the expression of *MyHC I* was upregulated (*P* < 0.05) in the probiotics group compared with that in the other three groups, and that of myogenic factor 6 (*Myf6*) was downregulated (*P* < 0.05) in the antibiotic group compared with that in the probiotics group (Figures 1A,B). At 95 days of age, the mRNA expression of *MyHC IIb* and myogenic factor 5 (*Myf5*) were upregulated (*P* < 0.05) in the probiotics and synbiotics groups compared to that in the control and antibiotic groups. The expression of *MyHC I* and *Myf6* were upregulated (*P* < 0.05) in the synbiotics group, whereas the expression of myostatin (*MSTN*) was downregulated (*P* < 0.05) when compared with the other three groups (Figures 1A,B). At 125 days of age, the expression of *Myf6* in the antibiotic, probiotics, and synbiotics groups and *MyHC IIa* in the antibiotic and synbiotics groups was upregulated (*P* < 0.05) compared with the control group. In addition, the expression of myogenin (*MyOG*) in the probiotics and synbiotics groups was downregulated (*P* < 0.05) compared to that in the control and antibiotic groups; moreover, the expression of *MyHC I* and *Myf5* were upregulated (*P* < 0.05) in the antibiotic group compared to that in the control and probiotics groups (Figures 1A,B).

As shown in Figures 1C,D, at 65 days of age, the expression of stearoyl-CoA desaturase (*SCD*) was downregulated (*P* < 0.05) in the antibiotic, probiotic, and synbiotics groups compared to that in the control group, whereas lipoprotein lipase (*LPL*) expression was upregulated (*P* < 0.05) in the probiotics group compared to that in the other three groups. At 95 days of age, the expression of *LPL* in the antibiotic, probiotics, and synbiotics groups and acetyl-CoA carboxylase (*ACC*) in the probiotics group were upregulated (*P* < 0.05) compared with the control group, and the expression of fatty acid synthase (*FASN*) in the probiotics and synbiotics groups and *SCD* in the synbiotics group were upregulated (*P* < 0.05) compared with the control and antibiotic groups. At 125 days of age, the expression of *LPL* was upregulated (*P* < 0.05) in the antibiotic, probiotics, and synbiotics groups compared with the control group. The expression of *ACC* was upregulated (*P* < 0.05) in the antibiotic and synbiotics groups and downregulated (*P* < 0.05) in the probiotics group compared with that in the control group. Moreover, the expression of *FASN* and *SCD* were upregulated (*P* < 0.05) in the antibiotic group compared with the other three groups, while the expression of hormone-sensitive triglyceride lipase (*LIPE*) in the antibiotic group was upregulated (*P* < 0.05) compared with that in the control group.

#### Plasma Biochemical Parameters

The effects of probiotics and synbiotics in sows' diets on the plasma biochemical parameters of the offspring are presented in Table 6. At 65 days of age, the plasma activity of α-AMS was lower in the antibiotic, probiotics, and synbiotics groups than in the control group, and LDH level was higher in the probiotics group and lower in the antibiotic group (*P* < 0.05). The plasma concentrations of LDL-C and TC were lower in the antibiotic group and higher in the synbiotics group than in the control group (*P* < 0.05). The plasma concentration of HDL-C was higher in the synbiotics group than in the other three groups, while GLU was lower in the control and probiotics groups (*P* < 0.05). The plasma concentrations of ALB in the antibiotic group and CHE in the probiotics group were lower (*P* < 0.05) than in the other three groups.

**Table 6:** Effects of probiotics and synbiotics addition in sows' diets on plasma biochemical parameters of offspring pigs.

**Items**	**Control**	**Antibiotic**	**Probiotics**	**Synbiotics**	**SEM**	***P-*values**
	**group**	**group**	**group**	**group**		
**ALT (U/L)**						
65 days of age	58.79	56.28	56.81	59.15	2.369	0.782
95 days of age	54.74^b^	54.22^b^	59.60^b^	78.33^a^	4.811	0.004
125 days of age	51.77	48.18	50.76	48.10	3.847	0.873
**ALP (U/L)**						
65 days of age	148.88	164.00	134.57	155.50	8.351	0.108
95 days of age	123.63	127.17	118.50	122.83	10.524	0.951
125 days of age	162.88^ab^	197.50^a^	142.83^b^	123.00^b^	13.740	0.010
**α-AMS (U/L)**						
65 days of age	2260.50^a^	1851.40^b^	1842.17^b^	1852.33^b^	76.887	0.001
95 days of age	2186.71	2074.40	2176.80	2271.60	80.523	0.405
125 days of age	2306.00^b^	2783.00^a^	2438.25^ab^	2806.00^a^	121.075	0.016
**AST (U/L)**						
65 days of age	59.75	56.40	73.71	53.83	6.181	0.129
95 days of age	73.30^a^	53.80^bc^	47.33^c^	63.75^ab^	3.606	<0.0001
125 days of age	65.17^a^	47.20^b^	49.50^b^	67.50^a^	5.292	0.023
**CHE (g/L)**						
65 days of age	534.63^a^	540.33^a^	420.29^b^	548.33^a^	16.605	<0.0001
95 days of age	516.25^a^	542.83^a^	427.60^b^	457.71^b^	15.986	<0.0001
125 days of age	467.67	475.25	445.50	487.25	13.724	0.203
**LDH (U/L)**						
65 days of age	416.75^b^	332.20^c^	503.67^a^	378.17^bc^	18.040	<0.0001
95 days of age	465.71^a^	374.43^b^	322.67^b^	437.67^a^	19.825	<0.0001
125 days of age	475.57^a^	383.25^b^	383.29^b^	411.25^ab^	26.291	0.042
**ALB (g/L)**						
65 days of age	41.23^a^	35.54^b^	41.21^a^	40.50^a^	0.643	<0.0001
95 days of age	40.45	39.46	40.58	40.48	0.657	0.596
125 days of age	43.86	44.53	45.18	43.93	0.915	0.677
**AMM (μmol/L)**						
65 days of age	167.93	159.24	203.50	189.87	14.293	0.137
95 days of age	265.37^a^	236.36^a^	171.86^b^	229.62^a^	17.090	0.005
125 days of age	332.90^a^	128.30^d^	198.58^c^	269.55^b^	21.299	<0.0001
**TP (g/L)**						
65 days of age	65.00	66.62	65.13	67.52	0.765	0.078
95 days of age	73.68^ab^	73.68^ab^	74.63^a^	70.82^b^	0.939	0.043
125 days of age	72.76^a^	68.90^b^	73.78^a^	73.10^a^	0.833	0.002
**UN (mmol/L)**						
65 days of age	3.10	3.20	3.18	2.87	0.341	0.901
95 days of age	3.56^a^	3.60^a^	2.42^c^	3.14^b^	0.127	<0.0001
125 days of age	3.47^ab^	3.98^a^	3.93^a^	3.35^b^	0.158	0.020
**HDL-C (mmol/L)**						
65 days of age	0.76^b^	0.75^b^	0.74^b^	0.88^a^	0.033	0.019
95 days of age	1.07	1.11	1.05	1.01	0.036	0.292
125 days of age	1.10^b^	1.22^a^	1.23^a^	1.09^b^	0.036	0.010
**LDL-C (mmol/L)**						
65 days of age	1.28^b^	1.08^c^	1.26^b^	1.50^a^	0.050	<0.0001
95 days of age	1.57	1.64	1.66	1.60	0.084	0.860
125 days of age	1.53	1.26	1.53	1.43	0.073	0.052
**TC (mmol/L)**						
65 days of age	2.37^b^	2.12^c^	2.31^b^	2.53^a^	0.053	<0.0001
95 days of age	2.68	2.90	2.85	2.83	0.073	0.199
125 days of age	2.78^ab^	2.59^b^	3.05^a^	2.78^ab^	0.082	0.004
**TG (mmol/L)**						
65 days of age	0.49	0.48	0.49	0.53	0.034	0.734
95 days of age	0.76^a^	0.60^ab^	0.63^ab^	0.51^b^	0.047	0.009
125 days of age	0.62^b^	0.61^b^	0.56^b^	0.82^a^	0.034	<0.0001
**GLU (mmol/L)**						
65 days of age	5.30^a^	5.12^ab^	5.30^a^	4.66^b^	0.176	0.049
95 days of age	6.40^a^	4.66^b^	5.18^b^	4.78^b^	0.246	<0.0001
125 days of age	4.48	4.92	4.66	4.80	0.161	0.261

*Data are presented as means with pooled SEM. Values in the same row with different superscript letters were significantly different (P < 0.05). The replicates per group at 65 and 95 days of age were 8; at 125 days of age, the replicates of the control group, antibiotic group, probiotics group, and synbiotics group were 8, 6, 8, and 6, respectively. ALT, alanine aminotransferase; ALP, alkaline phosphatase; α-AMS, α-amylase; AST, aspartate aminotransferase; CHE, cholinesterase; LDH, lactate dehydrogenase; ALB, albumin; AMM, ammonia; TP, total protein; UN, urea nitrogen; HDL-C, high-density lipoprotein-cholesterol; LDL-C, low-density lipoprotein-cholesterol; TC, total cholesterol; TG, triglyceride; GLU, glucose*.

At 95 days of age, the plasma activities of AST and LDH in the antibiotic and probiotics groups, GLU concentration in the antibiotic, probiotics, and synbiotics groups, and TG concentration in the synbiotics group were lower (*P* < 0.05) than in the control group. Plasma CHE activity and UN concentration were lower (*P* < 0.05) in the probiotics and synbiotics groups than in the control and antibiotic groups, while plasma AMM concentration was lower in the probiotics group and ALT concentration was higher in the synbiotics group than in the other three groups (*P* < 0.05).

At 125 days of age, plasma HDL-C concentration was higher (*P* < 0.05), and AST and LDH activities were lower (*P* < 0.05) in the antibiotic and probiotics groups than in the control group. The plasma AMM concentration was lower (*P* < 0.05) in the antibiotic, probiotics, and synbiotics groups, while α-AMS activity was higher (*P* < 0.05) in the antibiotic and synbiotics groups than in the control group. The plasma TP concentration in the antibiotic group was lower (*P* < 0.05), and the TG concentration in the synbiotics group was higher (*P* < 0.03) than in the other three groups.

#### Plasma Free Amino Acid Concentration

The effects of probiotics and synbiotics in sows' diets on plasma free AA concentrations in the offspring are presented in Table 7. At 65 days of age, the plasma His concentration in the probiotics group and β-aminoisobutyric acid (β-AiBA) concentrations in the antibiotic and synbiotics groups were higher (*P* < 0.05), whereas the plasma hydroxyproline (Hypro) concentration in the antibiotic, probiotics, and synbiotics groups, Gly, Ile, sarcosine (Sar), and taurine (Tau) concentration in the antibiotic and probiotics groups, and Leu concentration in the probiotics group were lower (*P* < 0.05) than in the control group. Compared with the control and antibiotic groups, the plasma citrulline (Cit) and cystathionine (Cysthi) concentrations were higher (*P* < 0.05) in the synbiotics group, whereas the plasma alanine (Ala) concentration was lower (*P* < 0.05) in the probiotics group. Plasma γ-amino-n-butyric acid (γ-ABA) concentration was higher in the synbiotics group than that in the other three groups, and plasma tyrosine (Tyr) concentration was lower, while the hydroxylysine (Hylys) concentration was higher in the probiotics group than in the other three groups (*P* < 0.05). However, the plasma threonine (Thr) and Pro concentrations were higher (*P* < 0.05) in the antibiotic group than in the other three groups.

**Table 7:** Effects of probiotics and synbiotics addition in sows' diets on plasma-free amino acid of offspring pigs (nmol/mL).

**Items**	**Control**	**Antibiotic**	**Probiotics**	**Synbiotics**	**SEM**	***P-*values**
	**group**	**group**	**group**	**group**		
**1-Mehis**						
65 days of age	0.85	0.95	0.50	0.90	0.147	0.157
95 days of age	6.13^b^	8.96^a^	5.06^b^	6.70^b^	0.626	0.001
125 days of age	0.93	0.89	0.44	1.16	0.201	0.095
**3-Mehis**						
65 days of age	12.38	11.64	11.69	12.13	0.766	0.885
95 days of age	14.09	13.74	12.71	15.27	0.730	0.123
125 days of age	13.92	15.41	17.17	16.37	1.082	0.146
**Ala**						
65 days of age	419.55^a^	445.57^a^	323.57^b^	391.40^ab^	24.723	0.011
95 days of age	358.50^a^	252.02^b^	296.22^ab^	347.86^a^	18.048	0.001
125 days of age	312.99^b^	393.20^a^	301.66^b^	284.56^b^	20.815	0.009
**Ans**						
65 days of age	1.05	1.04	0.98	0.88	0.105	0.682
95 days of age	0.48^a^	0.46^a^	0.51^a^	0.36^b^	0.028	0.005
125 days of age	22.53^c^	55.86^a^	35.01^b^	21.56^c^	2.124	<0.0001
**Arg**						
65 days of age	70.36	79.78	83.70	88.64	4.783	0.070
95 days of age	89.22^ab^	67.25^b^	69.73^b^	102.12^a^	6.953	0.004
125 days of age	84.84^b^	105.52^a^	85.15^b^	88.29^b^	3.540	0.001
**Asp**						
65 days of age	7.88	9.41	8.84	7.39	1.256	0.665
95 days of age	9.86	10.99	11.55	9.53	0.748	0.211
125 days of age	10.85^a^	12.44^a^	6.99^b^	10.34^a^	0.679	<0.0001
**Car**						
65 days of age	10.51	10.61	10.72	7.03	1.152	0.087
95 days of age	10.93^a^	8.97^a^	3.30^b^	8.24^a^	0.985	<0.0001
125 days of age	18.25	17.06	13.97	15.10	1.300	0.083
**Cit**						
65 days of age	25.65^c^	30.35^b^	23.54^c^	34.81^a^	1.272	<0.0001
95 days of age	40.77^a^	31.80^b^	30.24^b^	34.47^b^	1.829	0.002
125 days of age	38.50	34.22	40.66	34.89	2.474	0.229
**Cysthi**						
65 days of age	13.04^b^	7.55^c^	13.52^b^	18.79^a^	0.927	<0.0001
95 days of age	6.05^b^	6.69^b^	7.17^ab^	8.37^a^	0.437	0.006
125 days of age	9.97^b^	9.13^b^	10.88^ab^	13.89^a^	1.027	0.036
**Cys**						
65 days of age	9.56	11.19	8.58	12.69	1.062	0.052
95 days of age	94.43	93.56	59.16	70.89	14.719	0.264
125 days of age	12.55	11.11	7.17	12.87	1.872	0.115
**EOHNH2**						
65 days of age	0.27	0.31	0.25	0.28	0.031	0.558
95 days of age	24.52^b^	36.69^a^	0.46^c^	0.27^c^	2.086	<0.0001
125 days of age	5.09^a^	5.31^a^	5.90^a^	2.95^b^	0.522	0.006
**Glu**						
65 days of age	186.00	211.73	197.39	183.61	11.824	0.334
95 days of age	207.52	175.66	213.62	215.32	11.319	0.066
125 days of age	155.75^a^	133.41^b^	159.41^a^	134.23^b^	4.571	<0.0001
**Gly**						
65 days of age	525.00^a^	441.15^b^	417.84^b^	517.45^a^	22.405	0.003
95 days of age	470.34	472.11	484.09	560.27	29.847	0.129
125 days of age	566.31^b^	744.00^a^	517.49^b^	582.66^b^	20.194	<0.0001
**His**						
65 days of age	36.13^bc^	38.06^ab^	39.58^a^	35.05^c^	0.834	0.003
95 days of age	43.72^a^	38.14^b^	37.98^b^	40.04^ab^	1.406	0.026
125 days of age	44.72^a^	47.79^a^	39.29^b^	44.59^a^	1.074	<0.0001
**Hylys**						
65 days of age	1.08^b^	0.99^b^	1.77^a^	1.00^b^	0.144	0.001
95 days of age	0.55^b^	0.21^b^	0.31^b^	17.46^a^	0.545	<0.0001
125 days of age	20.27	17.00	16.29	13.96	2.598	0.404
**Hypro**						
65 days of age	80.45^a^	63.26^b^	67.49^b^	63.80^b^	3.157	0.002
95 days of age	37.70	27.34	31.12	40.87	3.707	0.062
125 days of age	67.51	73.25	61.61	56.72	4.385	0.098
**Ile**						
65 days of age	100.01^a^	89.39^b^	85.87^b^	106.56^a^	3.376	0.001
95 days of age	90.11^a^	72.10^b^	68.44^b^	75.76^b^	3.811	0.002
125 days of age	86.29^b^	98.37^ab^	95.59^ab^	101.40^a^	3.731	0.042
**Leu**						
65 days of age	164.89^a^	150.55^ab^	141.18^b^	157.55^ab^	4.946	0.015
95 days of age	140.61^a^	110.86^b^	108.49^b^	101.71^b^	6.546	0.001
125 days of age	142.49^c^	164.70^b^	145.71^c^	188.21^a^	5.000	<0.0001
**Lys**						
65 days of age	143.17	132.49	136.97	143.94	6.986	0.619
95 days of age	125.61	124.84	107.98	137.62	9.034	0.167
125 days of age	135.46^bc^	152.98^a^	124.49^c^	146.49^ab^	4.722	0.001
**Met**						
65 days of age	15.68	15.23	14.20	14.48	0.603	0.306
95 days of age	12.76	10.83	10.76	11.79	0.641	0.113
125 days of age	12.73^c^	16.16^a^	14.10^b^	12.84^c^	0.305	<0.0001
**Orn**						
65 days of age	51.91	55.78	59.34	56.87	3.443	0.500
95 days of age	56.21^a^	40.61^b^	53.07^a^	64.72^a^	4.032	0.002
125 days of age	68.98^a^	55.47^b^	43.97^b^	56.39^b^	3.736	<0.0001
**Phe**						
65 days of age	81.15	85.83	78.38	79.57	3.381	0.436
95 days of age	81.64	79.95	73.05	83.18	2.810	0.077
125 days of age	90.72^ab^	92.96^a^	85.38^b^	88.08^ab^	1.540	0.009
**Pro**						
65 days of age	173.12^b^	214.35^a^	163.76^b^	188.85^b^	7.593	<0.0001
95 days of age	157.10	143.26	154.65	148.96	8.298	0.650
125 days of age	183.79^b^	224.84^a^	162.65^b^	160.26^b^	11.179	0.002
**Sar**						
65 days of age	8.53^a^	3.72^b^	4.13^b^	6.10^ab^	0.875	0.002
95 days of age	0.41^c^	0.66^c^	6.67^a^	2.08^b^	0.381	<0.0001
125 days of age	2.25	2.38	2.28	2.28	0.115	0.855
**Ser**						
65 days of age	75.51	87.29	82.69	80.79	3.228	0.101
95 days of age	81.57	75.69	78.11	75.35	2.589	0.318
125 days of age	81.30^b^	97.57^a^	69.65^c^	73.61^c^	1.802	<0.0001
**Tau**						
65 days of age	133.56^a^	108.96^bc^	100.33^c^	121.25^ab^	5.264	0.001
95 days of age	138.40^a^	116.37^b^	116.01^b^	116.55^b^	5.698	0.022
125 days of age	137.88^b^	153.35^a^	133.68^b^	128.73^b^	5.074	0.021
**Thr**						
65 days of age	116.70^b^	151.48^a^	108.88^b^	117.32^b^	7.145	0.001
95 days of age	105.91	107.08	113.80	114.86	8.913	0.852
125 days of age	119.87^b^	147.49^a^	111.62^b^	113.26^b^	4.004	<0.0001
**Tyr**						
65 days of age	50.03^a^	47.53^a^	24.34^b^	47.37^a^	4.762	0.002
95 days of age	44.93	53.06	48.90	52.60	2.562	0.112
125 days of age	62.07^b^	67.95^a^	60.31^b^	60.48^b^	1.552	0.008
**Val**						
65 days of age	249.16^ab^	222.48^b^	220.34^b^	264.19^a^	12.257	0.047
95 days of age	213.22	183.85	205.35	209.81	8.517	0.089
125 days of age	237.37^b^	283.85^a^	257.01^ab^	284.97^a^	13.022	0.044
**α-AAA**						
65 days of age	65.81	56.64	67.93	57.40	4.768	0.247
95 days of age	60.05^a^	45.61^b^	47.63^b^	45.07^b^	2.733	0.002
125 days of age	55.04^ab^	49.07^ab^	57.68^a^	44.23^b^	3.369	0.049
**α-ABA**						
65 days of age	15.47^b^	24.10^a^	15.68^b^	18.36^ab^	2.277	0.042
95 days of age	12.16^a^	4.47^b^	0.66^c^	4.87^b^	1.147	<0.0001
125 days of age	2.68^c^	3.45^b^	3.70^ab^	4.06^a^	0.131	<0.0001
**β-Ala**						
65 days of age	8.33	5.87	6.00	7.30	0.763	0.096
95 days of age	8.88^a^	3.91^c^	2.70^c^	6.33^b^	0.555	<0.0001
125 days of age	6.91^b^	8.60^a^	6.97^b^	7.85^ab^	0.273	<0.0001
**β-AiBA**						
65 days of age	0.52^b^	1.43^a^	0.33^b^	1.35^a^	0.084	<0.0001
95 days of age	0.66^c^	2.55^a^	1.72^b^	1.73^b^	0.196	<0.0001
125 days of age	0.61^b^	1.05^a^	0.59^b^	0.37^c^	0.054	<0.0001
**γ-ABA**						
65 days of age	0.25^b^	0.21^b^	0.16^b^	0.59^a^	0.041	<0.0001
95 days of age	0.67^a^	0.23^b^	0.18^b^	0.16^b^	0.060	<0.0001
125 days of age	1.81	1.89	1.66	1.64	0.115	0.374

*Data are presented as means with pooled SEM. Values in the same row with different superscript letters were significantly different (P < 0.05). The replicates per group at 65 and 95 days of age were 8; at 125 days of age, the replicates of the control group, antibiotic group, probiotics group, and synbiotics group were 8, 6, 8, and 6, respectively. 1-Mehis, 1-methyl-histidine; 3-Mehis, 3-methyl-histidine; Ala, alanine; Ans, anserine; Arg, arginine; Asp, aspartate; Car, carnosine; Cit, citrulline; Cysthi, cystathionine; Cys, cysteine; EOHNH2, ethanolamine; Glu, glutamate; Gly, glycine; His, histidine; Hylys, hydroxy-lysine; Hypro, hydroxy-proline; Ile, isoleucine; Leu, leucine; Lys, lysine; Met, methionine; Orn, ornithine; Phe, phenylalanine; Pro, proline; Sar, sarcosine; Ser, serine; Tau, taurine; Thr, threonine; Tyr, tyrosine; Val, valine; α-AAA, α-aminoadipic acid; α-ABA, α-amino-n-butyric acid; β-Ala, β-alanine; β-AiBA, β-aminoisobutyric acid; γ-ABA, γ-amino-n-butyric acid*.

At 95 days of age, the plasma Ile, Leu, Cit, Tau, α-aminoadipic acid (α-AAA), α-amino-n-butyric acid (α-ABA), β-alanine (β-Ala), and γ-ABA concentrations were lower, and β-AiBA was higher in the antibiotic, probiotics, and synbiotics groups than in the control group (*P* < 0.05). Compared with the control and antibiotic groups, plasma Sar concentration was higher, while EOHNH2 concentration was lower in the probiotics and synbiotics groups, and plasma Cysthi concentration was higher in the synbiotics group (*P* < 0.05), while plasma Hylys concentration was higher, whereas anserine (Ans) concentration was lower in the synbiotics group than in the other three groups (*P* < 0.05). However, the Plasma 1 methyl-histidine (1-Mehis) and EOHNH2 concentrations were higher, whereas orthenine (Orn) concentration was lower in the antibiotic group than in the other three groups (*P* < 0.05).

At 125 days of age, the plasma Ans and methionine (Met) concentrations in the antibiotic and probiotics groups and the valine (Val) and Leu concentrations in the antibiotic and synbiotics groups were higher (*P* < 0.05) than in the control group. Plasma α-ABA concentration was higher, whereas Orn concentration was lower in the antibiotic, probiotics, and synbiotics groups than in the control group (*P* < 0.05). Compared with the control and antibiotic groups, plasma Cysthi concentration was higher in the synbiotics group, while Ser concentration was lower in the probiotics and synbiotics groups (*P* < 0.05). The plasma Asp and His concentrations in the probiotics group, and the EOHNH2 concentration in the synbiotics group were lower (*P* < 0.05) than in the other three groups; however, plasma Ala, Arg, Gly, Pro, Ser, Tau, Thr, Tyr, and β-AiBA concentrations were higher (*P* < 0.05) in the antibiotic group than in the other three groups.

## Discussion

There is growing scientific and industrial interest in the addition of prebiotics, probiotics, and synbiotics to pig feed (28, 29). Numerous studies have shown that dietary probiotics and synbiotics can improve the health and production performance of pigs by modulating their intestinal microbiota and metabolites (30, 31). Therefore, the present study determined the effects of maternal gut microbiota intervention *via* dietary antibiotic, probiotics, or synbiotics on the growth performance, carcass traits, meat quality, and metabolism of offspring. Our findings indicate that the addition of probiotics and synbiotics to Bama mini-pig diets could improve the feed intake of offspring pigs and meat quality by increasing water-holding capacity and cooking yield, enhancing meat tenderness and sense-impression, and regulating metabolism and related gene expression.

Probiotics and synbiotics are widely used in livestock production. Consistent with the fact that dietary *L. plantarum* ZLP001 addition could increase the ADFI of weaning piglets (32), our results showed that the addition of maternal probiotics increased the ADFI in 66–95-day-old piglets. Recent studies have found that dietary probiotics supplementation can improve growth performance by increasing the final BW, ADG, and G/F in growing-finishing Landrace × Yorkshire × Talent pigs (33); however, neither maternal probiotics nor synbiotics addition affected the ADG and F/G of the offspring of Bama mini-pigs in the present study. A possible reason for this inconsistency may be related to pig breeds. Bergamaschi et al. (34) reported that three pig breeds had different feed efficiencies because of their different gut microbiota compositions. In addition, the present study demonstrated that maternal antibiotic addition significantly decreased the ADFI of offspring pigs between 66 and 95 days of age and increased the F/G of those between 96 and 125 days of age, which may be related to the adverse effects of antibiotic use during pregnancy in piglets (35). A previous study also indicated that maternal antibiotic addition could alter the maternal and fetal gut microbiota, thereby affecting the health of offspring (36). Therefore, the adverse effects of maternal antibiotic addition on the growth performance of offspring require further investigation.

Carcass traits and meat quality are the major factors that influence meat flavor, tenderness, juiciness, and overall consumer acceptance. In the present study, the addition of maternal probiotics and synbiotics increased the backfat thickness and fat percentage of offspring pigs at 125 days of age along with the addition of antibiotic, while probiotics addition increased backfat thickness at 65 days of age, suggesting that these additives could increase body fat deposition and improve meat tenderness in 125-day-old pigs. Generally, the loin-eye area is positively related to lean meat rate and negatively related to backfat thickness; however, the present study showed a decrease in loin-eye area and lean meat rate in the synbiotics group at 125 days of age, indicating that maternal synbiotics had a negative impact on lean meat rate. These results are consistent with those of previous studies that found that dietary XOS addition had no significant effect on the lean meat rate of Landrace pigs (37, 38). This inconsistency may be due to pig breed, as the Bama mini-pig is a fatty breed with a generally higher fat content than Landrace pigs (26).

Meat color is an important sensory index because it affects consumers' first impression of meat (39). High-quality meat has higher redness and lower lightness and yellowness (40). In the present study, maternal synbiotics addition increased the redness values at 95 days of age and decreased the lightness values at 125 days of age in the LT muscle of the offspring; maternal probiotics addition decreased the lightness at 65 days of age and redness values at 95 days of age in the LT muscle. However, Meng et al. (39) indicated that dietary probiotics addition could increase redness values but not lightness values, and Cheng et al. (4) reported that dietary synbiotics (including yeast cell wall, XOS, *Clostridium butyricum, B. licheniformis*, and *B. subtilis*) had no effect on redness and lightness values. These differences may be related to feeding stage, type, and dose of probiotics or synbiotics. Although maternal synbiotics addition partially improved the sense-impression of pork, the addition of maternal probiotics had no positive effect on meat color. However, further studies are needed to determine the exact reason for this finding.

After slaughter, the accumulation of lactic acid in the muscle due to glycolysis leads to decrease in pH, which is highly correlated with drip loss and shear force (41). Drip loss can reflect the water-holding capacity of muscle and is an important factor affecting meat quality (42), whereas shear force is correlated with meat tenderness (43). Previous studies have reported that the addition of *L. plantarum* ZJ316 to the diet of piglets improved meat quality by increasing pH_45min_ value (44), and that *B. coagulans* addition affected meat quality by decreasing drip loss (45). Consistent with the above mentioned studies, our results showed that maternal probiotics addition increased pH_45min_ value at 95 days of age and cooking yield at 65 days of age. Moreover, maternal probiotics and synbiotics addition decreased the drip loss at 65 days of age and shear force at 125 days of age. These findings suggest that the addition of maternal probiotics could decrease lactic acid accumulation by improving muscle glycolysis, and that the addition of maternal probiotics and synbiotics could increase water-holding capacity and cooking yield by reducing drip loss, cooking loss, and shear force, thereby improving meat quality. Maternal antibiotic addition had a negative effect on meat quality because of increased shear force of the LT muscle at 125 days of age.

Changes in the nutrient composition of muscular tissue, especially IMF and CP content, can directly affect the sensory properties and nutritional value of meat (46). The tenderness, juiciness, color, and flavor of meat are substantially improved with increase in IMF content (47). Moreover, higher IMF content can improve the taste of meat (43). In the present study, maternal synbiotics addition increased the IMF content of the LT muscle at 125 days of age, which is consistent with the change in shear force, because IMF content has a negative correlation with it (48). These findings suggest that maternal synbiotics supplementation can improve meat quality by improving its tenderness.

The composition of AAs in muscles can represent the protein quality and nutritional value of the meat (49). The present study showed that dietary probiotics and synbiotics addition significantly increased the TAA content in the LT muscle at 95 days of age, suggesting that the nutritional value of pork was improved by increasing AA deposition. Tang et al. (50) also reported that dietary supplementation with *B. subtilis* improved the meat protein quality and flavor of broilers. Several AAs play key roles in the aroma and flavor profiles of muscles (51). For example, Arg, Leu, Ile, Val, Phe, Met, and His induce a bitter taste; glutamate (Glu) and Asp induce a pleasant fresh taste; and Gly, Ala, and Ser induce a sweet taste (52). Our results showed that maternal probiotics addition increased His content at 65 days of age, whereas maternal synbiotics addition increased His and Ser content at 125 days of age in the LT muscle of the offspring pigs. Moreover, maternal probiotics and synbiotics addition increased Arg and Asp contents at 95 days of age, maternal probiotics addition increased Leu content, and maternal synbiotics addition increased Phe content at 125 days of age in the LT muscle of the offspring pigs, which could improve its flavor. Consistent with our findings, a previous study indicated that dietary *C. butyricum* addition improved the flavor of duck meat by altering the content of flavor-determining AAs (53).

Free AAs are the main direct source of AAs for muscle protein synthesis and are a key indicator of protein renewal (54). The plasma-free AA profile reflects the sum of the metabolic flow of nutrients and their metabolites in all tissues and organs (55). For example, Gly plays crucial roles in nutrition and metabolism, and protein synthesis accounts for 80% of the whole-body glycine requirement of growing animals (56). Branched-chain AAs, including Leu, Ile, and Val, affect the regulation of energy homeostasis and nutritional metabolism (57). In the present study, the addition of maternal probiotics decreased plasma Gly, Ile, and Leu concentrations at 65 days of age, and the addition of probiotics and synbiotics decreased plasma Ile, Leu, and Tau at 125 days of age, suggesting that protein breakdown was attenuated and AA deposition was promoted in the muscles. In addition, maternal probiotics supplementation decreased plasma Ala, Sar, and Tyr concentrations at 65 days of age, Car, His, and α-ABA concentrations at 95 days of age, and Asp, Ala, His, α-AAA, and β-Ala concentrations at 125 days of age, while probiotics and synbiotics addition decreased plasma Cit, α-AAA, β-Ala, β-AiBA, and γ-ABA concentrations at 95 days of age and Orn concentration at 125 days of age, suggesting that protein synthesis in the body was improved. However, the underlying mechanism requires further investigation.

ALT and AST activities can reflect the status of protein synthesis and catabolism, and increased activity is related to improvement of AA metabolism (58). AMM and UN concentrations accurately reflect the status of protein metabolism and AA balance. In the present study, maternal synbiotics addition increased ALT activity, while maternal probiotics addition decreased AMM concentration at 95 days of age. Maternal probiotics and synbiotics addition decreased UN concentration at 95 days of age and AMM concentration at 125 days of age, suggesting that AA balance and protein utilization in pigs were improved. These findings are consistent with the changes in plasma-free AA observed in the present study.

The rate of lipid utilization is reflected in TC, TG, HDL-C, and LDL-C levels (59). The plasma levels of LDH and GLU reflect energy and glucose metabolism, respectively. In the present study, maternal synbiotics addition increased HDL-C, LDL-C, and TC concentrations at 65 days of age and decreased TG concentration at 95 days of age; however, maternal antibiotic addition decreased the concentrations of LDL-C and TC at 65 days of age. Similarly, a previous study indicated that dietary supplementation with synbiotics composed of XOS, yeast cell wall, *B. licheniformis, B. subtilis*, and *C. butyricum* could regulate lipid metabolism by increasing HDL-C level and decreasing TG level in chickens (60). In addition, LDH activity was decreased in the probiotics group at 95 and 125 days of age, as well as GLU concentration in the synbiotics group at 65 days of age and in the probiotics and synbiotics groups at 95 days of age. These findings suggest that the addition of maternal probiotics and synbiotics improved lipid and glucose metabolism in the offspring, and that antibiotic addition had a negative effect on lipid metabolism.

FA composition profoundly affects the nutritional value, organoleptic properties, and eating quality of pork (61, 62). Our study showed that maternal probiotics addition increased C18:2n6c content at 65 days of age and MUFA and UFA contents at 95 days of age in the LT muscle. This was consistent with a previous study, which found that the addition of dietary *L. amylovorus* and *Enterococcus faecium* could increase muscular C18:2n6c, MUFA, and PUFA contents (63). These alterations indicate that the addition of maternal probiotics could improve the primary FA content in the muscle of offspring, which may be associated with favorable changes in the gut microbiota (64). The FA profiles of pork can be improved by increasing the UFA and decreasing the SFA content in the muscle (63). In the present study, the addition of maternal probiotics decreased the SFA content at 65 days of age, and the addition of probiotics and synbiotics increased the C16:l content at 95 days of age. These findings indicate that these additives could improve the nutritional value of meat and pork flavor of pigs, because SFA is negatively correlated with cardiovascular heart diseases (65), while C16:l content is positively correlated with meat flavor (66). In addition, the addition of maternal probiotics and synbiotics decreased the C18:1n9t content at 125 days of age. C18:1n9t is a trans-FA that has adverse effects on human health (67). Consequently, the addition of maternal probiotics and synbiotics can improve the meat quality and nutritional value of the offspring.

Muscle fiber type is mainly defined by MyHC isoforms (68), and fiber type composition is largely responsible for the determination of meat quality (69). Our study showed that maternal probiotics and synbiotics addition upregulated the expression of *MyHC IIa*, and that maternal probiotics addition upregulated the expression of *MyHC I* in 65-day-old pigs. Moreover, maternal synbiotics addition upregulated the expression of *MyHC I* in 95-day-old pigs and *MyHC IIa* in 125-day-old pigs, whereas it downregulated the expression of *MyHC IIb* and *MyHC IIx* in 65-day-old pigs. Higher proportion of *MyHC I* and *MyHC IIa* fibers is associated with excellent meat quality (70). The MRF gene family, including *MyoD, MyoG, Myf5*, and *Myf6*, plays a dominant role in the formation, maturation, and functional perfection of muscle fibers (71), the expression of which affects the expression of MyHC gene isoforms (72). *MSTN*, a negative regulator of myogenesis, plays an important role in muscle fiber conversion (73) and inhibits muscle growth and development (74). In the present study, the addition of maternal probiotics and synbiotics upregulated *Myf5* and *Myf6* mRNA level at 95 and 125 days of age, respectively, whereas synbiotics addition downregulated *MSTN* mRNA level at 95 days of age. Tian et al. (75) also showed that the dietary probiotic *L. reuteri* 1 upregulated the expression of *MyHC I* and *MYOD* and downregulated the expression of *MyHC IIb*. These findings suggest that the addition of maternal probiotics and synbiotics had positive effects on enhancing muscle growth and altering muscle fiber type composition.

Lipogenesis and lipolysis are major factors that affect fat accumulation in the muscle and adipose tissues. *FASN* and *ACC* modulate fat deposition by regulating FA synthesis (26), and *SCD* is involved in FA biosynthesis and composition in animals (76). In the present study, the addition of maternal probiotics and synbiotics upregulated *FASN* mRNA level in 95-day-old pigs and *ACC* mRNA level in 65- and 95-day-old pigs. Maternal synbiotics addition upregulated *SCD* mRNA level in 95-day-old pigs and *ACC* mRNA level in 125-day-old pigs in the LT muscle, suggesting that fat deposition was improved. In addition, the addition of maternal probiotics and synbiotics downregulated *SCD* mRNA level in 65-day-old pigs. However, further studies are required to clarify the exact mechanisms of action. *LPL*, a critical lipid uptake gene, encodes a protein that participates in the process of fatty acid flux in adipocytes clustered along myofiber fasciculi in muscles and then provides an appropriate substrate for IMF synthesis (77). The expression of *FASN, SCD*, and *LPL* is positively associated with IMF deposition (78). Our results demonstrate that the addition of maternal probiotics and synbiotics had beneficial effects on IMF deposition by upregulating *LPL* mRNA expression at 95 and 125 days of age. This was consistent with the increased IMF content in the LT muscle in the present study. Moreover, *LIPE* participates in lipolysis and is negatively correlated with IMF deposition (78). Our results also showed that maternal antibiotic addition upregulated the expression levels of *FASN, LPL, SCD, ACC*, and *LIPE* in the offspring at 125 days of age, suggesting that antibiotic addition has adverse effects on lipid metabolism in the LT muscle. These findings are consistent with those of a previous study, which reported that *L. reuteri* 1 was maintained IMF by inducing higher lipogenic rate and lower lipolytic rate than antibiotics (75).

## Conclusion

In summary, the addition of maternal synbiotics improved feed intake, whereas that of antibiotic showed adverse effects on the feed intake of the during at 66–95 days of age. Maternal probiotics and synbiotics can increase the meat quality of offspring by improving the composition of muscle fibers, growth, and nutrient metabolism. However, maternal antibiotic addition had adverse effects on lipid metabolism of the LT muscle of the offspring pigs at different stages. Collectively, the addition of maternal probiotics and synbiotics, as a nutritional intervention strategy, improved the feed intake and meat quality in some contexts by altering metabolism and gene expression related to the meat quality of offspring pigs.

## Data Availability Statement

The original contributions presented in the study are included in the article/Supplementary Material, further inquiries can be directed to the corresponding author/s.

## Ethics Statement

The animal study was reviewed and approved by the Animal Care and Use Committee of the Institute of Subtropical Agriculture, Chinese Academy of Science.

## Author Contributions

XK and YY conceived and designed the experiments and revised the manuscript. QZ, MS, MA, and CM performed the animal feeding experiments, sample collection, and sample analysis. QZ analyzed the data and wrote the manuscript. All the authors have read and approved the final version of the manuscript.

## Funding

This study was jointly supported by the National Natural Science Foundation of China (31772613), Special Funds for Construction of Innovative Provinces in Hunan Province (2019RS3022), and the Industry and Research Talent Support Project of Wang Kuancheng of the Chinese Academy of Sciences.

## Conflict of Interest

The authors declare that the research was conducted in the absence of any commercial or financial relationships that could be construed as a potential conflict of interest. The reviewer JJ declared a shared affiliation with the authors to the handling editor at the time of review.

## Publisher's Note

All claims expressed in this article are solely those of the authors and do not necessarily represent those of their affiliated organizations, or those of the publisher, the editors and the reviewers. Any product that may be evaluated in this article, or claim that may be made by its manufacturer, is not guaranteed or endorsed by the publisher.
