# Supplementary material for: Probiotics and Synbiotics Addition to Bama Mini-Pigs' Diet Improve Carcass Traits and Meat Quality by Altering Plasma Metabolites and Related Gene Expression of Offspring
Source: Front Vet Sci. 2022 Jul 5;9:779745. doi: 10.3389/fvets.2022.779745 (PMC9301501; doi:10.3389/fvets.2022.779745)
Supplement: Supplementary file 1 [file Data_Sheet_1.PDF]

Supplementary Table 1

Composition and nutrient levels of basal diets for sows (air-dry basis; %).

Items	Pregnant diet	Lactating diet
Ingredients		
Corn	37.50	66.00
Soybean meal	9.50	25.00
Wheat bran	14.00	5.00
Barley	25.00	
Soybean hull	10.00	
Pregnant premix[1)]	4.00	
Lactating premix[2)]		4.00
Total	100.00	100.00
Nutrient levels[3)]		
Digestible energy (MJ/Kg)	12.55	13.87
Crude protein	12.82	16.30
Crude fiber	4.56	2.87
SID[4)] Lys	0.48	0.75
SID Met+ Cys	0.43	0.51
SID Thr	0.37	0.53
SID Trp	0.13	0.17
Calcium	0.62	0.65
Phosphorus	0.47	0.50

[1)] Pregnant premix provided the following per kg of diet: $CaHPO_4 \cdot 2H_2O$ 10 g, NaCl 4 g, $CuSO_4 \cdot 5H_2O$ 80 mg, $FeSO_4 \cdot H_2O$ 360 mg, $ZnSO_4 \cdot H_2O$ 240 mg, $MnSO_4 \cdot H_2O$ 100 mg, $MgSO_4 \cdot 7H_2O$ 1 g, 1% ICl 50 mg, 1% Na_2SeO_3 36 mg, 1% $CoCl_2$ 16 mg, $NaHCO_3$ 1.4 g, VA 10 000 IU, VD_3 1 800 IU, VE 20 mg, VK_3 2.4 mg, VB_1 1.6 mg, VB_2 6 mg, VB_6 1.6 mg, VB_{12} 0.024 mg, folic acid 1.2 mg, nicotinamide 20 mg, pantothenic acid 12 mg, biotin 0.12 mg, ferrous glycinate 100 mg, choline chloride 1g, phytase 200 mg, fruity 80 mg, limestone 12 g.

[2)] Lactating premix provided the following per kg of the diet: $CaHPO_4 \cdot 2H_2O$ 10 g, NaCl 4 g, $CuSO_4 \cdot 5H_2O$ 80 mg, $FeSO_4 \cdot H_2O$ 360 mg, $ZnSO_4 \cdot H_2O$ 240 mg, $MnSO_4 \cdot H_2O$ 100 mg, 1% ICl 50 mg, 1% Na_2SeO_3 36 mg, 1% $CoCl_2$ 16 mg, $NaHCO_3$ 1.4 g, VA 10 000 IU, VD_3 1 800 IU, VE 20 mg, VK_3 2.4 mg, VB_1 1.6 mg, VB_2 6 mg, VB_6 1.6 mg, VB_{12} 0.024 mg, folic acid 1.2 mg, nicotinamide 20 mg, pantothenic acid 12 mg, biotin 0.12 mg, Lysine 1.5 g, ferrous glycinate 100 mg, choline chloride 1g, phytase 200 mg, fruity 80 mg, limestone 12 g.

[3)] Nutrient levels were calculated values.

[4)] SID: standard ileum digestible.

Supplementary Table 2

Composition and nutrient levels of basal diets for weaned Bama mini-pigs (air-dry basis; %).

Items	Prophase diet (35−95 days of age)	Anaphase diet (96−125 days of age)
Ingredients		
Corn	54.92	58.00
Soybean meal	22.00	18.35
Wheat bran	10.13	11.35
Rice bran	8.95	8.30
Premix[1)]	4.00	4.00
Total	100.00	100.00
Nutrient levels[2)]		
Digestible energy (MJ/kg)	13.50	13.42
Crude protein	16.13	14.90
Calcium	0.45	0.44
Total Phosphorus	0.49	0.49
Lys	1.40	1.30
Met + Cys	0.69	0.66
Thr	0.78	0.74

[1)] Premix provided the following per kilogram of diets: enzyme preparation (including phytase, protease, and lipase) 1.2 g, VA 26 000 IU, VD_3 10 000 IU, VE 70 IU, VK_3 10 mg, VB_1 10 mg, VB_2 25 mg, VB_6 10 mg, VB_{12} 0.075 mg, biotin 0.4 mg, folic acid 5 mg, nicotinamide 100 mg, pantothenic 50 mg, choline 1600 mg, flavoring agent 500 mg, edulcorant 300 mg, acidulating agent 5 g, $CuSO_4 \cdot 5H_2O$ 898 mg, $MnSO_4 \cdot H_2O$ 298 mg, $ZnSO_4 \cdot H_2O$ 600 mg, $FeSO_4 \cdot H_2O$ 501 mg, $Ca(IO_3)_2$ 0.9 mg, as Na_2SeO_3 0.7 mg, $CoSO_4 \cdot H_2O$ 1.2 mg, glucose 2.1 g, antioxidants 0.4 g, anti-mildew agent 1 g, Ca (as $CaHPO_4$ and $CaCO_3$) 3.42 g, P (as $CaHPO_4$) 1.155 g.

[2)] Nutrient levels were calculated values.

Supplementary Table 3

Primers sequences used for real time-PCR

Genes	Sequence (5’ to 3’)	Size (bp)	GenBank ID
β-actin	F: GGCACCACACCTTCTACAACGAG<br>R: TCATCTTCTCACGGTTGGCTTTGG	102	XM_021086047.1
ACC	F: CAAAGAGGTTCCAGGCACAGTCC<br>R: CGTCAGCATGTCAGAAGGCAGAG	146	NM_001114269.1
FASN	F: TACCTTGTGGATCACTGCATAGA<br>R: GGCGTCTCCTCCAAGTTCTG	113	NM_001099930.1
LIPE	F: GCGTGCTCTCCAAGTGTGTCAG<br>R: CCAGGCGGAGGTCTCGGAAG	135	NM_214315.3
LPL	F: ACACAGTTGAGGACACTTGCCATC<br>R: TCCTGTCACCGTCCAGCCATG	116	NM_214286.1
Myf5	F: GGATCAGCAACTCCGAGCAACC<br>R: GCACATGGTAGATGAGCCTGGAAC	126	NM_001278775.1
Myf6	F: GCTCGTGATGACTGCCAAGGAAG<br>R: CGATGGAAGAAAGGCACCGAAGG	80	NM_001244672.1
MyoG	F: AAACTACCTGCCCGTCCACCTC<br>R: GGTCCCCAGCCCCTTATCTTCC	112	NM_001012406.1
MSTN	F: GCACCAAGCAAACCCCAGAGG<br>R: AGCACCCACAGCGATCTACTACC	143	NM_214435.2
MyHC I	F: CTGTCCAAGTTCCGCAAGGT<br>R: CTTTGTTGCGCCCTCAGGAT	176	NM_213855.2
MyHC IIa	F: GGACCCCCTGAATGACACAG<br>R: CGGTCTGGAAGGAAGAACCC	149	NM_214136.1
MyHC IIx	F: TGAGGAAGACCGCAAGAACA<br>R: GGTCACTTTTGAGCATTTGGATG	272	NM_001104951.2
MyHC IIb	F: AGGAGCATCAGCGCCTAATC<br>R: TCGGGATAGCTGAGACACCA	119	NM_001123141.1
SCD	F: ATTGGGAGCTGTGGGTGAG<br>R: AAGTTGATGTGCCAGCGGTA	90	NM_213781.1
SREBP-1	F: GCGACGGTGCCTCTGGTAGT<br>R: CGCAAGACGGCGGATTTA	218	NM_214157.1

ACC, acetyl-CoA carboxylase; *FASN*, fatty acid synthase; *LIPE*, hormone-sensitive triglyceride lipase; *LPL*, lipoprotein lipase; *Myf5*, myogenic factor 5; *Myf6*, myogenic factor 6; *MyOG*, myogenin; *MSTN*, myostatin; *SCD*, stearoyl-CoA desaturase; *SREBP-1*, sterol-regulatory element-binding protein-1.
